# Efficacy and safety of a bio‐absorbable antibiotic delivery in calcium sulphate granules for the treatment of osteomyelitis in patients with diabetic foot: A randomized, double blinded, controlled clinical study The BIG D‐FOOT study

**DOI:** 10.1111/dom.16254

**Published:** 2025-02-19

**Authors:** Matteo Monami, Laura Bordoni, Benedetta Ragghianti, Giovanni Antonio Silverii, Edoardo Mannucci

**Affiliations:** ^1^ Diabetology, Careggi Hospital Florence Italy; ^2^ University of Florence Florence Italy

**Keywords:** cost‐effectiveness, diabetes complications, diabetic neuropathy, randomized trial

## Abstract

**Background:**

Diabetic foot osteomyelitis (DFO) is associated with a considerably high risk of incident major amputations, disability and mortality. To assess the effects of a local antibiotic delivery system on the incidence of post‐surgical infective complications in patients with DFO.

**Methods:**

This is a double‐blind, placebo‐controlled, parallel series, randomized controlled trial (RCT) aimed at verifying the efficacy and safety of a local calcium‐sulphate bio‐absorbable antibiotic delivery (either with tobramycin or vancomycin) in patients with DFO treated with surgical procedures. The trial enrolled adult patients with diabetes and Texas 3 grade ulcers complicated by osteomyelitis and accompanied by deep tissues infection. The primary end‐point was the percentage of infective complications (composite end‐point of dehiscence, infection, DFO recurrence and new DFO in adjacent sites) at 12 weeks.

**Results:**

The study was prematurely terminated after the completion of the first 20 cases, because of the significant superiority of the active treatment arm. After 12 weeks of treatment, five of 20 wounds (25%) achieved the primary composite end‐point. All post‐surgical infective complications occurred in the placebo group, with a significant between‐group difference (unadjusted *p* = 0.010). No between‐group differences in overall costs were observed.

**Conclusions:**

This is the first RCT in patients with DFO showing that the use of antibiotic‐impregnated calcium‐sulphate granules is economically sustainable and has the potential of improving the prognosis of DFO.

## 1 INTRODUCTION

Diabetic foot infections are a frequent complication of diabetic foot ulcers (DFU), which are associated with a higher mortality and lower‐limb amputation rate.^1^, ^2^, ^3^, ^4^ In particular, diabetic foot osteomyelitis (DFO), deep infections involving the bone, usually require adequate surgical debridement procedures, which are prone to complications, and a prolonged antimicrobial therapy. Systemic antibiotic use, however, is often challenging, due to concomitant peripheral artery disease, which might impair drug distribution to tissues; furthermore, comorbid conditions such as renal or liver insufficiency may reduce tolerability to antibiotics, whereas the selection of antibiotic‐resistant bacterial strains may reduce their efficacy.^5^, ^6^, ^7^, ^8^

During the last two decades, several biodegradable carriers capable of locally releasing antibiotics have been developed: proteins (collagen, gelatin, thrombin, etc.), synthetic polymers, grafts and substitutes (calcium sulphate or phosphate). These innovative devices could overcome some of the above‐mentioned barriers to antibiotic treatment, reducing the risk of post‐surgical infections and accelerating healing processes, but available evidence is still limited, and mainly represented by observational data.^9^, ^10^, ^11^, ^12^ A few randomized trials published on skeletal infections and infected non‐unions using different carriers, such as antibiotic‐impregnated bone grafting^13^ and beads in orthopaedic patients,^9^, ^14^, ^15^ were performed several years ago with encouraging results.

Calcium sulphate granules (Stimulan Rapid Cure™) were shown to reduce adverse outcomes both in patients with infected DFU and DFO in non‐randomized small‐sized studies providing promising results.^10^, ^16^, ^17^, ^18^, ^19^, ^20^ To our knowledge, no randomized trial was ever performed to date to verify the efficacy of calcium sulphate granules as a carrier for local antibiotic delivery in patients with DFO.

The present study is designed as a double‐blind, placebo‐controlled, parallel series, randomized trial aimed at verifying the efficacy and safety of a local calcium‐sulphate bio‐absorbable antibiotic delivery (either with tobramycin or vancomycin) in patients with DFO treated with surgical procedures. The study was prematurely terminated after the completion of the first 20 cases, because the difference in the incidence of the primary end‐point between the active treatment and control groups was already significant.

## 2 MATERIALS AND METHODS

The present paper reports the results of a prospective double‐blind, placebo‐controlled trial evaluating post‐surgical infective complications rates of DFO treated with a local bio‐absorbable antibiotic delivery in calcium sulphate granules in an outpatient setting.

The trial was designed to verify whether, in patients with DFO undergoing surgical therapy, being assigned to the insertion of a local bio‐absorbable antibiotic in calcium sulphate granules delivery would reduce the incidence of post‐surgical infections, in comparison with being assigned to the insertion of calcium sulphate granules alone.

The Area Vasta Centro Ethical Local Committee approved both the study protocol and informed consent form on 17 October 2023 (No. 23487_spe). The study was registered at ClinicalTrials.gov (NCT06262854; https://ichgcp.net/clinical-trials-registry/nct06262854) and conducted at Careggi Teaching Hospital in Florence in adherence to Good Clinical Practice guidelines and the Declaration of Helsinki on 25th February 2024. This trial was reported following the CONSORT guidelines.^21^

### 2.1 Patients screening and eligibility

The trial enrolled adult patients with diabetes (both type 1 or type 2 diabetes) and ulcers penetrating to bone or joint (Texas 3 grade ulcers^22^), complicated by osteomyelitis and accompanied by deep tissues infection, and located in the forefoot. We only included forefoot osteomyelitis to reduce possible confounding factors, because the site of the ulcer and osteomyelitis may influence the overall prognosis.^23^ A detailed list of inclusion and exclusion criteria is reported in Supporting Information (Table S1). The diagnosis of DFO was established on the basis of a combination of probe‐to‐bone test and plain X‐ray showing bone destruction, cortical reabsorption, periosteal reaction or a sequestrum.^24^

### 2.2 Study procedures

A complete medical history was collected at enrollment; for each patient, Charlson's comorbidity score^25^ was calculated to assess the burden of comorbidities. The duration of the wound (i.e., the time occurred from the onset of the wound) and the ulcer location were recorded.

Samples were drawn for biochemistry and bacteriological analysis of index lesions. Appropriate procedures were applied for the assessment of arteriopathy and neuropathy (See ‘Study procedures’ in Data S1).

All patients underwent minor amputations (i.e., finger amputation and/or metatarsal osteotomy) and ulcerectomy; following the standard of care of our unit, all amputations were performed in an ambulatory setting of a trained diabetologist (MM). Surgical procedures were carried out after local regional anaesthesia was performed with 10 cc lidocaine 2%. Necrotic tissues, pus and infected soft tissues were removed until bleeding. If osteomyelitis was located in the diaphysis, devitalized bones at the base of ulcers were exposed and excised to the level of healthy cancellous and cortical bone. If possible, the bases of the metatarsal and phalangeal bones were preserved for healthy tendons attaching. When infection was located in interphalangeal or metatarsophalangeal joints, both the joints and partial distal and proximal bones were excised. Fibrous tissues, fascia and tendons nearby were also completely removed. Following bone resection, irrigations with a solution of iodopovidone were performed.

### 2.3 Randomization and blinding

A computer‐generated random number generator was used to randomize (1:1) patients into the following two groups: Local Bio‐Absorbable Antibiotic Delivery (LBAAD) or matching placebo. A researcher not involved in the trial procedure prepared the randomization list, and allocation was performed via telephone calls. In cases in which the results of the biopsy and culture specimens complete with antibiograms showed that isolated germs were equally sensitive to both antibiotics, a further randomization list between vancomycin and tobramycin was performed with the same procedure as above. Calcium‐sulphate granules used for the two groups were identical and researchers and patients were unable to distinguish the two treatments. Other therapeutic procedures after the application of the investigational treatments are reported in Data S1 (See ‘Other therapeutic procedures’ in Data S1).

### 2.4 Treatments

In both treatment arms, according to international guidelines,^26^ empiric oral antibiotic therapy (either Levoxacin or Cefepime) was prescribed before surgical intervention; when the result of the biopsy was available, antibiotic therapy was adjusted according to the results of culture specimens. In any case, antibiotic therapy was stopped after 72 h from surgical intervention, unless clinical signs and symptoms of infections were not resolved.

At the end of the above‐mentioned surgical procedure (see para. 2.3), patients allocated to LBAAD received antibiotic‐impregnated calcium‐sulphate granules prepared during the surgical procedures in an adjacent operating room by an unblinded operator not involved in any other trial procedures. On the basis of antibiogram results, patients received either vancomycin or tobramycin. In case of microorganisms responsive to both antibiotics, a further randomization was performed between the two antibiotics. The antibiotic chosen was mixed into the synthetic calcium‐sulphate (Stimulan Rapid Cure™, Biocomposite Ltd., UK) with a recommended ratio: 500 mg vancomycin with 5 mL calcium‐sulphate or 120 mg tobramycin with 5 mL calcium‐sulphate, dissolved in a sterile saline solution and injected into the dead space (Canada‐STIMULAN‐Brochure‐MA0033R3.pdf).

Patients allocated to the placebo arm received with matched calcium‐sulphate granules without antibiotics, following the same procedures.

In case of DFO recurrence after surgery, antibiotic therapy was administered following international guidelines.^27^

### 2.5 End‐points

The efficacy primary end‐point was the percentage of infective complications (composite end‐point of dehiscence, infection, DFO recurrence and new DFO in adjacent sites) at 12 weeks. Recurrence of DFO was defined as the onset of osteomyelitis in the same or an adjacent site, after complete healing of the DFU that was the point of entry of the infection and the surgical wound.^28^ Secondary end‐points included individual components of the primary end‐point, and other efficacy end‐points, that is, new onset of osteomyelitis in other foot sites (not adjacent to the index DFO), reintervention for abscesses drainage or recurrent DFO, lower‐limb major amputation, proportion of healed patients, surgical wound time‐to‐healing, direct medical costs and quality of life, whereas serious and non‐serious adverse events (SAE and AE) and all‐cause death were the safety end‐points.

Dehiscence was defined as any infective process (Perfusion, Extent, Depth, Infection and Sensation—PEDIS >1) in the surgical site within 7 days from surgical procedures. Infective complications were defined as any infective process (PEDIS >1^29^) in the surgical site that occurred between 7 days after surgical procedure and the end of the study. Complete healing, defined as full epithelialization of the wound with the absence of drainage, was recorded at post‐surgical visits. Time to healing was defined as the number of days that occurred from the surgical procedure to complete re‐epithelialization. Serious adverse events were defined as any event or adverse reaction which corresponds to one or more of the following criteria: (a) fatal outcome; (b) is life‐threatening; (c) requires hospitalization or determines a prolongation of it. Major amputation was defined as a surgical procedure performed above the ankle. Quality of Life (QoL) was measured using the Short Form‐12 (SF‐12).^30^

### 2.6 Economic assessment

An economic descriptive analysis was performed considering the perspective of the local health system, thus considering only direct healthcare costs and including costs associated with healthcare resources use all through the follow‐up and extracted from clinical records as reported in the Data S1 (See ‘Economic assessments’ in Data S1).

### 2.7 Statistical analyses

The sample size was determined to be 50 (25 in each group) to achieve 90% power to detect a difference between the group proportions of 0.33 in the incidence of infective post‐surgical complications (dehiscence, infection, DFO recurrence and new DFO in adjacent sites), which were expected to occur in 25% of treated patients, as reported in our previous article.^1^ An unblinded interim analysis was planned after completion of the first 20 subjects, in order to eventually recalculate the sample size for the primary end‐point or to prematurely interrupt the study for safety/efficacy reasons (between‐group differences with *p*‐values ≤0.010). All the analyses were based on the intention to‐treat principle, thus including all patientswho underwent randomization.^31^

Chi‐square test (with *p* < 0.05) was used for between‐group comparison of categorical variables, whereas non‐categorical variables (i.e., SF‐12) were compared using the Mann–Whitney *U* test. A Kaplan–Meyer analysis was performed to assess time‐dependent effects of the two treatments on the primary end‐point, using a log rank test.^32^

Statistical analysis was performed on SPSS 25.0. Data were expressed as mean ± standard deviation (Std. dev), or as median (25th–75th percentile), depending on their distribution.

## 3 RESULTS

Out of 52 screened patients, 20 were eligible to be randomized either to LBAAD and placebo, resulting in 10 subjects per each treatment group (Figure S1). Of those, two patients in the intervention army, and one patient in the comparator army were women, respectively. No patients were lost to follow‐up and two patients in the placebo group underwent a below‐knee amputation. No deaths were recorded. Patient‐ and wound‐related parameters at baseline were well‐balanced between the two groups, as shown in Table 1. Microbiological findings are summarized in Table 1.

**TABLE 1:** Wound‐ and patient‐related parameters between the two groups at randomization.

Parameter	LBAAD (*n* = 10)	Placebo (*n* = 10)	*p*
Women (%)	2 (20)	1 (10)	0.55
Patients' characteristics			
Age (years)	67.5 ± 11.1	70.5 ± 13.9	0.64
Diabetes duration (years)	20 (12;24)	24 (10;41)	1.00
Body mass index (kg/m^2^)	27.5 ± 7.1	28.3 ± 7.9	0.71
HbA1c (mmol/mol)	72.2 ± 35.1	63.5 ± 19.5	0.52
Serum creatinine (mg/dL)	0.9 (0.7;1.4)	1.1 (0.8;2.3)	0.62
eGFR (mL/min)	64.2 (43; 84)	53 (31.5; 79.2)	0.35
Insulin treatment (*n*, %)	6 (60)	6 (60)	1.00
Peripheral artery disease (*n*, %)	5 (50)	6 (60)	0.59
Neuropathy (*n*, %)	8 (80)	8 (80)	1.00
Charlson's comorbidity score	3 (3;4.7)	4 (2.2;5.7)	1.00
Short Form‐12			
Physical Component Summary	40 (30; 44)	34 (30; 39)	0.38
Mental Component Summary	42 (36; 60)	45 (27; 51)	0.50
**Antibiotic used**			
Tobramycin	5	‐	‐
Vancomycin	5	‐	‐
**Foot examination**			
TcPO_2_ at the allux (mmHg)	36 (32; 46)	30 (27; 43)	0.19
Ankle‐Brachial Index	0.9 (0.7; 1.5)	0.9 (0.7; 1.3)	0.97
Pain (VAS, 1–10 points)	1.5 (0; 3.5)	0.5 (0; 3.2)	0.80
Wound duration (days)	218 (152; 310)	172 (57; 266)	0.25
Wound location (*n*, %)			0.58
Toe	6 (60)	7 (70)	
Metatarsal	3 (30)	3 (30)	
Both	1 (10)	0 (0)	
Wound plantar surface (*n*, %)	3 (30)	2 (20)	0.61
Wound severity (*n*, %)			1.00
TUC 3B	2 (20)	2 (20)	
TUC 3D	8 (80)	8 (80)	
**Antibiogram results** (*n*, %)			
Patients with >1 microorganisms	6 (60)	1 (10)	0.019
Gram‐positive organisms	9	3	0.006
S. aureus	3	2	0.61
Enterococcus	3	1	0.14
Streptococci	2	0	0.15
Corynebacterium	1	0	0.30
Gram‐negative organisms	6	9	0.12
Proteus	1	2	0.61
Tobramicyn resistance	2	0	0.14
Vancomycin resistance	6	8	0.33
Pseudomonas	3	4	0.64
Klebsiella	1	1	1.00
Enterobacter	0	0	‐
*E. coli*	0	1	0.30
Morganella	0	1	0.30
Citrobacter	0	1	0.30
Stenotrophomonas maltophila	1	0	0.30

*Note*: Data are presented as percentage, mean ± SD and median (interquartile). TUC: Texas' University Classification.

All patients received an initial systemic treatment with levofloxacin.

**FIGURE 1:**
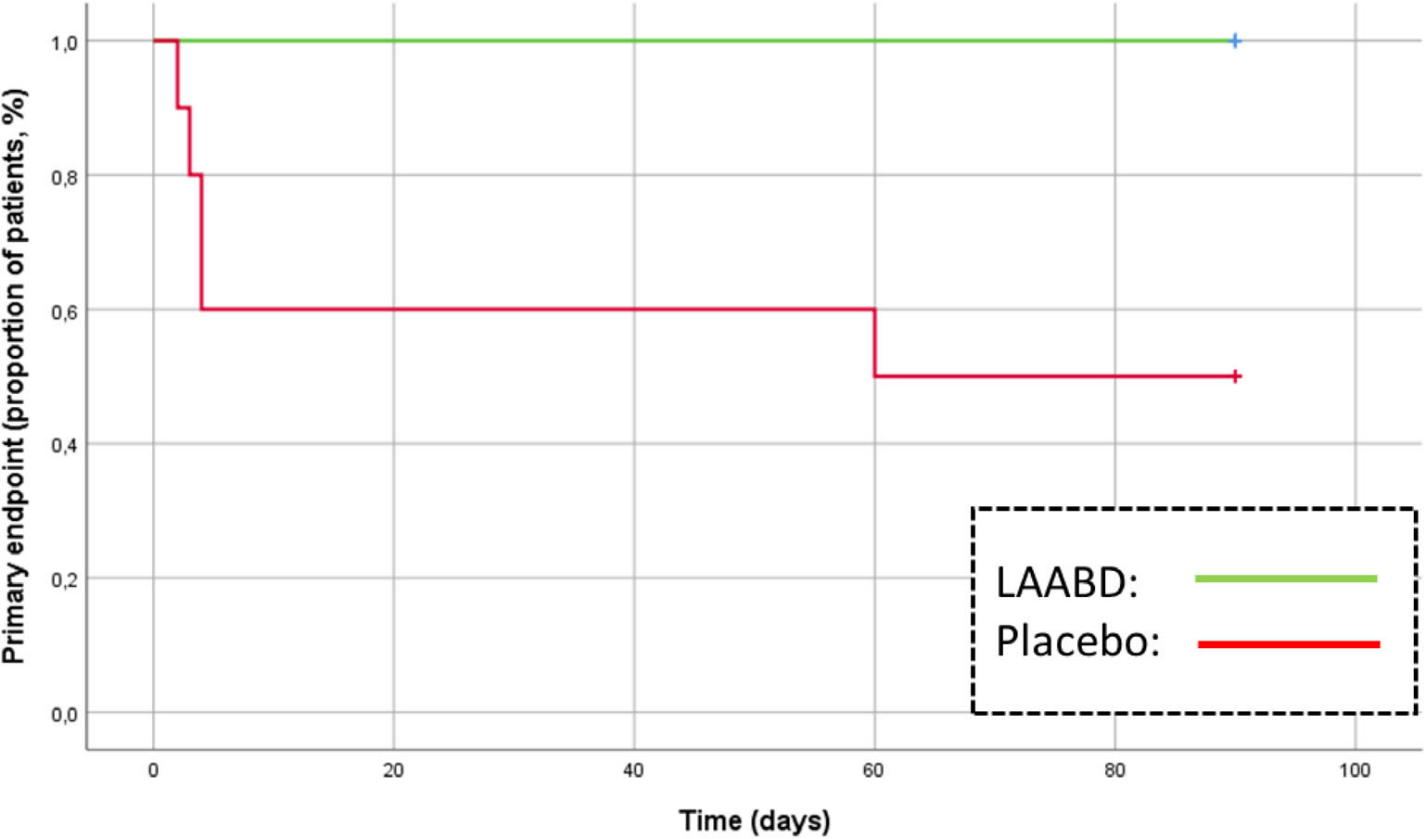
Proportion of patients experiencing post‐surgical infective complications (primary composite end‐point) during the 90‐day follow‐up (*p* = 0.010). Green line: Local Bio‐Absorbable Antibiotic Delivery (LBAAD); Red line: placebo.

After 12 weeks of treatment, five of 20 wounds (25%) achieved the primary composite end‐point. All post‐surgical infective complications occurred in the placebo group (the majority of which within the first 2 weeks), with a significant between‐group difference (unadjusted *p* = 0.010) at a Kaplan–Meyer analysis (Figure 1). The same results were observed for dehiscence/infection (five cases vs. zero in the placebo and LBAAD arm, respectively; Table 2).

**TABLE 2:** Description of categorical secondary end‐points.

Outcome (*n*)	LBAAD	Placebo	RR, 95% CI	*p*
Dehiscence/Infection	0/10	5/10	‐	0.010
DFO recurrence	0/10	2/10	‐	0.26
Major amputation^a^	0/10	2/10	‐	
DFO in non‐adjacent sites	0/10	0/10	‐	‐
New surgical intervention	0/10	1/10	‐	0.48
^a^	0/10	2/10	‐	0.26
Ulcer healing	5/10	3/10	2.33 [0.37; 14.6]	0.36

^a^ These two events were caused by post‐surgical infective complications.

Three SAEs (a DFO recurrence after 2 weeks confirmed by plain‐RX, and two major amputations after 15 and 25 days) were observed in the placebo arm requiring intravenous antibiotics. No patients allocated to the intervention arm required systemic antibiotic therapy. Out of 20 post‐surgical plain‐RX performed after 90 days as per local protocol, only two showed signs of DFO recurrence. No further plain‐RX alterations have been observed in the remaining patients. The patient in the placebo group who experienced a DFO recurrence underwent a new surgical procedure (after breaking the blinding code), using the device with antibiotics (tobramycin) and achieving a complete healing after 2 months. The other planned secondary end‐points are summarized in Tables 2 and 3, with no statistical between‐group significance, except for *SF‐12 Physical component*, which showed significantly better results in favour of LBAAD (*p* = 0.038 between groups, see Table 3).

**TABLE 3:** Description of other efficacy and safety secondary end‐points, including serious and non‐serious adverse events.

Outcome	LBAAD	Placebo	*p*
Time‐to‐healing	71 ± 26	78 ± 26	0.57
Antibiotic therapy duration (days)	11.9 ± 5.2	20.1 ± 11.9	0.060
SF‐12 Physical component	40 (36.2; 51.4)	21 (24.9; 40)	**0.038**
SF‐12 Mental component	47.4 (40.5; 58)	35.5 (25.5; 57.1)	0.19
Total direct foot‐related costs	2384 ± 113	4003 ± 6063	0.41
Stimulan Rapid Cure™ 5 mL costs	1987 ± 0	0	‐
Visits costs	169 ± 62	171 ± 97	0.96
Hospital admission costs	0	3384 ± 5725	0.076
Systemic antibiotic therapy costs	36 ± 15	193 ± 243	0.057
Laboratory/imaging exams costs	193 ± 82	256 ± 143	0.24
Any non‐serious adverse events	3/10	3/10	1.00
Ischaemic dehiscence	1	0	
New ulceration	2	1	
Mild hypertension	0		
Mild hyperglycaemia	0	1	
Serious adverse events	0/10	2*/10	0.26
Below‐knee amputation	0	2	
DFO recurrence	0	2	
All‐cause death	0/10	0/10	‐

*Note*: Patients with DFO recurrence both underwent a below‐knee major amputation; LBAAD, Local Bio‐Absorbable Antibiotic Delivery. Data are presented as percentage, mean ± SD, median (interquartile) and Odds Ratio (95%, Confidence Intervals; OR, 95% CI).

No between‐group differences in overall costs (median and mean) during the 90‐day follow were observed, as shown in Table 3.

## 4 DISCUSSION

DFO is a challenging condition for clinicians involved in the management of diabetic foot ulcers. The penetration of antibiotics in the bone is often difficult, usually achieving insufficient therapeutic concentrations, often because of concomitant peripheral vascular disease. Moreover, comorbid conditions, such as renal insufficiency, limit the long‐term use of many systemic antibiotics.

Several different local delivery systems have been explored in order to overcome these problems, such as antibiotic‐impregnated collagen sponges, with beneficial effects on the healing rate of infected moderately‐severely infected diabetic foot ulcers, without bone involvement, in randomized clinical trials.^19^, ^33^, ^34^, ^35^ To our knowledge, no RCTs have been performed in patients with DFO assessing these antibiotic‐impregnated devices. Only uncontrolled studies have been performed in patients with DFO using other similar local delivery systems (e.g., carriers with protein or synthetic polymers, grafts, etc.).^36^

Calcium‐sulphate granules, which have not been assessed through randomized trials similarly to the other devices mentioned above, could have some advantages in comparison with other local delivery systems^37^ (e.g., carriers with protein or synthetic polymers, grafts, etc.) in fact, calcium.sulfate granules are biodegradable, and they also show predictable elution properties. Other possible advantages of calcium‐sulfate granules include osteoconductivity, and the capability to fill dead space.^20^

We show here for the first time in a placebo‐controlled randomized trial conducted in a sample of patients with DFO and deep soft tissue infection that antibiotic‐impregnated calcium‐sulphate granules are capable of reducing post‐surgical infective complications. The efficacy of this treatment was confirmed by the reduction of dehiscence/infection and DFO recurrence in the LAABD arm, and by the improvement of the physical domain of health‐related quality of life.

The strengths of the present study include a robust trial design, the double‐blind nature, which is often very difficult to apply to studies on diabetic foot, a standardized and structured approach to standard of care.^38^, ^39^, ^40^ Another strength is the width of observed difference between the two treatment arms, which led to the premature termination of the trial.

At the same time, the interruption of the study after the first interim analysis prevented the enrollment of the full planned sample of 50 patients, limiting the statistical power of the analyses on many secondary end‐points. Notably, the power calculation for establishing a correct sample size had been performed assuming the occurrence of the primary end‐points in 25% of subjects in the control group, based on previous observations^10^; the end‐point was actually observed in five out of 10 enrolled patients in the control group. Considering the limited sample size, the difference between the observed and expected incidence of the primary end‐point in the control group could be a play of chance. However, it cannot be excluded that the application of calcium sulphate granules without antibiotics favours bacterial superinfection. This consideration induced the investigators, after consulting the local Ethical Board, to terminate the trial as soon as a significant superiority of the treatment arm for the primary end‐point was achieved. A possible detrimental impact of ‘placebo’ (i.e., calcium sulphate without antibiotics) would produce an overestimation of treatment effects; therefore, the efficacy of LAABD should be confirmed through open‐label randomized trials, accepting possible biases derived from the open label design.

Further limitations of the study are its monocentric nature and the exclusion of some categories of patients such as those with severe renal impairment and peripheral artery disease, which limit the generalizability of results. In addition, the principal end‐point included events based on the investigators' judgement, which could be operator‐dependent. Furthermore, the limited sample size could have prevented an adequate balance in characteristics of patients between treatment and control group, with the former showing a higher proportion of polymicrobic Gram+ infections, and a trend towards a more preserved renal function; any possible subgroup analyses were also prevented. The small number of treated subjects also limits the reliability of safety assessments; in fact, it cannot be ruled out that, in a larger sample of patients, cases of post‐surgical infections would also occur in the treatment arm. The trial was designed with a short follow‐up (12 weeks), in order to include only post‐surgical infections, reducing the risk of misclassifications (i.e., cases of new infections). As expected, and in line with the available literature,^41^ the majority of the events occurred within the first 2 weeks (Figure 1).

The use of new devices and techniques is often limited by high costs. However, the cost of the device is only a small part of direct costs for the clinical management of DFO, which include antibiotics, hospital admissions, specialist visits, laboratory examinations, etc.^42^ For this reason, we performed an analysis of direct costs actually recorded in the two treatment groups, showing no significant difference between antibiotic‐impregnated calcium‐sulphate granules and traditional approaches. In fact, the cost of the device was more than compensated by savings for antibiotics and other direct costs (hospital admission, major amputations and number of visits) and with a possible amelioration of patients' quality of life probably related to an earlier functional recovery.

Despite the limitations listed above, this randomized trial obtained promising results, suggesting that the use of antibiotic‐impregnated calcium‐sulphate granules might be economically sustainable and has the potential of improving the prognosis of diabetic foot osteomyelitis. Further randomized, open‐label trials are needed to confirm these results and collect greater information on possible adverse events.

## AUTHOR CONTRIBUTIONS

BR and MM designed the study and contributed to data collection, interpretation and writing. AS and LB contributed to data collection and writing the manuscript. EM contributed to writing and supervised the data quality control. MM is the guarantor of this work and takes responsibility for the integrity of the data and the accuracy of the data analysis.

## FUNDING INFORMATION

The authors received no financial support for the research, authorship and/or publication of this article.

## CONFLICT OF INTEREST STATEMENT

The authors declared no potential conflict of interest with respect to the research, authorship and/or publication of this article.

### PEER REVIEW

The peer review history for this article is available at https://www.webofscience.com/api/gateway/wos/peer-review/10.1111/dom.16254.

## ETHICS STATEMENT

This study was submitted to local Ethical Committee (Area Vasta Centro, Firenze) and received formal approval (Protocol number 23487_spe).

## Supporting information


**DATA S1:** Supporting Information.

## Data Availability

The data that support the findings of this study are available on request from the corresponding author. The data are not publicly available due to privacy or ethical restrictions.
