# Supplementary material for: Efficacy and safety of a bio‐absorbable antibiotic delivery in calcium sulphate granules for the treatment of osteomyelitis in patients with diabetic foot: A randomized, double blinded, controlled clinical study The BIG D‐FOOT study
Source: Diabetes Obes Metab. 2025 Feb 19;27(5):2552–60. doi: 10.1111/dom.16254 (PMC11964986; doi:10.1111/dom.16254)

**SUPPLEMENTARY MATERIALS**

**Efficacy and safety of a Bio-absorbable antibiotic delivery in calcium sulfate Granules for the treatment of osteomyelitis in patients with Diabetic FOOT: a randomised, double blind, controlled clinical study.** *The BIG D-FOOT Study.*

Matteo Monami; Laura Bordoni; Benedetta Ragghianti; Giovanni Antonio Silverii; Edoardo Mannucci.

***Study procedures***

All subjects underwent a 14-day screening period following the initial visit. At the first visit, following an established standard procedure of the Clinic, a full physical examination was undertaken collecting demographic parameters and a full-detailed medical history including information on concomitant medications, complications of diabetes, comorbid conditions, and laboratory exams (i.e., serum creatinine and glycosylated haemoglobin (HbA1c), and haemoglobin).

A full evaluation of current wounds was then conducted and when multiple DFOs were eligible on a subject's feet, the largest wound was selected. Ulcer area was measured only in case of multiple wounds in order to decide which one had to be included in the study. No further analyses regarding ulcer size and its follow-up have been performed.

In all patients, a tissue biopsy with a scalpel or punch biopsy instrument, following Levine technique, was taken for bacteriological analysis^25^. As per local standard of care, transcutaneous pressure of oxygen (TcpO2; Radiometer Medical ApS; Brønshøj, Denmark) at the basis of the first toe and amputation performed only for values >25 mmHg.

Screening of PAD was performed by assessing wave form analysis and measuring ABI and/or TBI; an Doppler Ultra Sound (DUS) examination of lower-limb arteries was performed when one or more screening tests was positive^26^. Diagnosis of diabetic neuropathy was performed by measuring vibratory perception threshold with a biothesiometer (METEDA, San Benedetto del Tornto, Italy) and a 10g monofilament^27^. Ulcers were classified according to the University of Texas score^28^.

***Other therapeutic procedures***

Local dressing used immediately after the surgical procedure consist of iodopovidone-impregnated gauze to be removed three times a week. After the first week the local dressing could be modified by the investigators involved in this trial. At each follow-up visit the wound was cleaned and sharply debrided, if required. Therapeutic shoes (with pressure-relieving insoles) were used in all patients with plantar ulcers, for pressure relief, as per the standard procedure of the Clinic. Subjects in both groups had weekly follow-up visits and dressing changes following the standard of care for up to 12 weeks. At each visit, wound sites were assessed for healing status, pain, and infection; the index ulcer was measured and AEs were reported. Plain X-ray was repeated in all patients at 90 days to evaluate the eradication of osteomyelitis. Quality of Life (QoL) was measured at visit V-2 and at the endpoint.

***Economic assessment***

Direct costs included specialist visits, diagnostic procedures, hospital admissions (related to diabetic foot), major and minor amputations, antibiotic therapy, grafts, and off-loading orthesis (Table 2S and 3S).

Costs for hospitalizations were estimated on the basis of established regional tariffs (https://www.salute.gov.it/portale/temi/p2_6.jsp id=3662&area=programmazioneSanitariaLea&menu=vuoto), i.e. tariffs established for the diagnosis-related group (DRG) associated with each episode for hospital admissions (either day-hospital or full-length stay) and recorded in clinical records; similarly for costs related to specialistic visits and outpatient procedures performed (e.g. RX, MRI, laboratory exams, etc.). The cost of antibiotic therapy was estimated considering ex-factory prices (https://www.salute.gov.it/portale/temi/p2_6.jsp?id=3662&area=programmazioneSanitariaLea&menu=vuoto),while current market prices were used to value costs for orthopaedic shoes/orthesis. The health economic analysis performed tried to estimate costs born to the healthcare system, mainly using tariffs related to different healthcare services, over one year. As discounting typically require the collection of data over different time point to give a different value to both costs and health outcomes that are predicted to occur in the future because they are usually valued less than present costs, given the time frame considered in our analysis we decided to do not apply any discount rate. All costs were referred to 2023.

**Table 1S** – Inclusion and exclusion criteria.

| **Inclusion criteria** | **Exclusion criteria** |
| --- | --- |
| 1. age≥ 18 years; 2. diagnosis of diabetes mellitus; 3. Texas 3 grade ulcers; 4. Diagnosis of osteomyelitis; 5. Deep tissues infection; 6. Ability to sign the written informed consent and attend the appointments during the follow-up period. 7. Ulcer located in the forefoot. Osteomyelitis located in metatarsal or phalangeal bones. | 1. Sepsis; 2. Severe peripheral arterial disease (Transcutaneous oximetry< 25 mmHg and/or Ankle Brachial Index< 0.5, and/or toe pressure <30 mmHg, and/or toe brachial index <0.35 and/or requiring an immediate revascularization); 3. Charcot foot; 4. Pregnancy; 5. Antibiotic allergies; 6. Estimated glomerular filtration rate< 30 ml/min; 7. Liver insufficiency; 8. Severe cognitive impairment. |

**Table 2S –** Costs (€) for hospital admission for foot-related conditions.

| **DRG code and description of procedure** | **Hospital length >1 day** | **Day-hospital** |
| --- | --- | --- |
| 114 – Toe amputation for vascular diseases | 8,962 | 731 |
| 205 – Lower limb amputation for metabolic or endocrinological disease vascular diseases | 13,431 | 482 |
| 130 – Peripheral revascularization with multiple comorbid conditions | 4,904 | 390 |
| 131 – Peripheral revascularization without multiple comorbid conditions | 3,398 | 390 |
| 556 – Peripheral revascularization with drug-eluting stent | 10,097 | 731 |

§ Hospital length without any additional costs; *additional daily costs for hospitalization > length threshold

**Table 3S –** Costs associated to procedures and laboratory examinations

| **DRG code and description of procedures** | **Costs (€)** |
| --- | --- |
| 88.28 Foot/ankle X-ray | 21 |
| 88.38.7 Foot/ankle computed tomography | 173 |
| 88.77.2 Lower limbs ecocholor doppler | 49.5 |
| 88.94.1 Foot/ankle Nuclear Magnetic Resonance | 254 |
| 88.42.2 Lower limbs arteriography (TC) | 283,3 |
| 89.65.4 Transcutaneous oxygen monitoring | 18.6 |
| 90.16.3 Creatinine | 1.2 |
| 90.28.1 Glycated Hemoglobin | 10.6 |
| 90.27.1 Glycemia | 1.30 |
| 90.14.3 Total cholesterol | 1.1 |
| 90.14.1 HDL cholesterol | 1.8 |
| 90.43.2 Triglycerides | 1.3 |
| 90.72.3 C-reactive protein | 3.6 |
| 86.11 Cutaneous biopsy | 13.9 |

**Figure 1S** – Flow chart of trial participants.


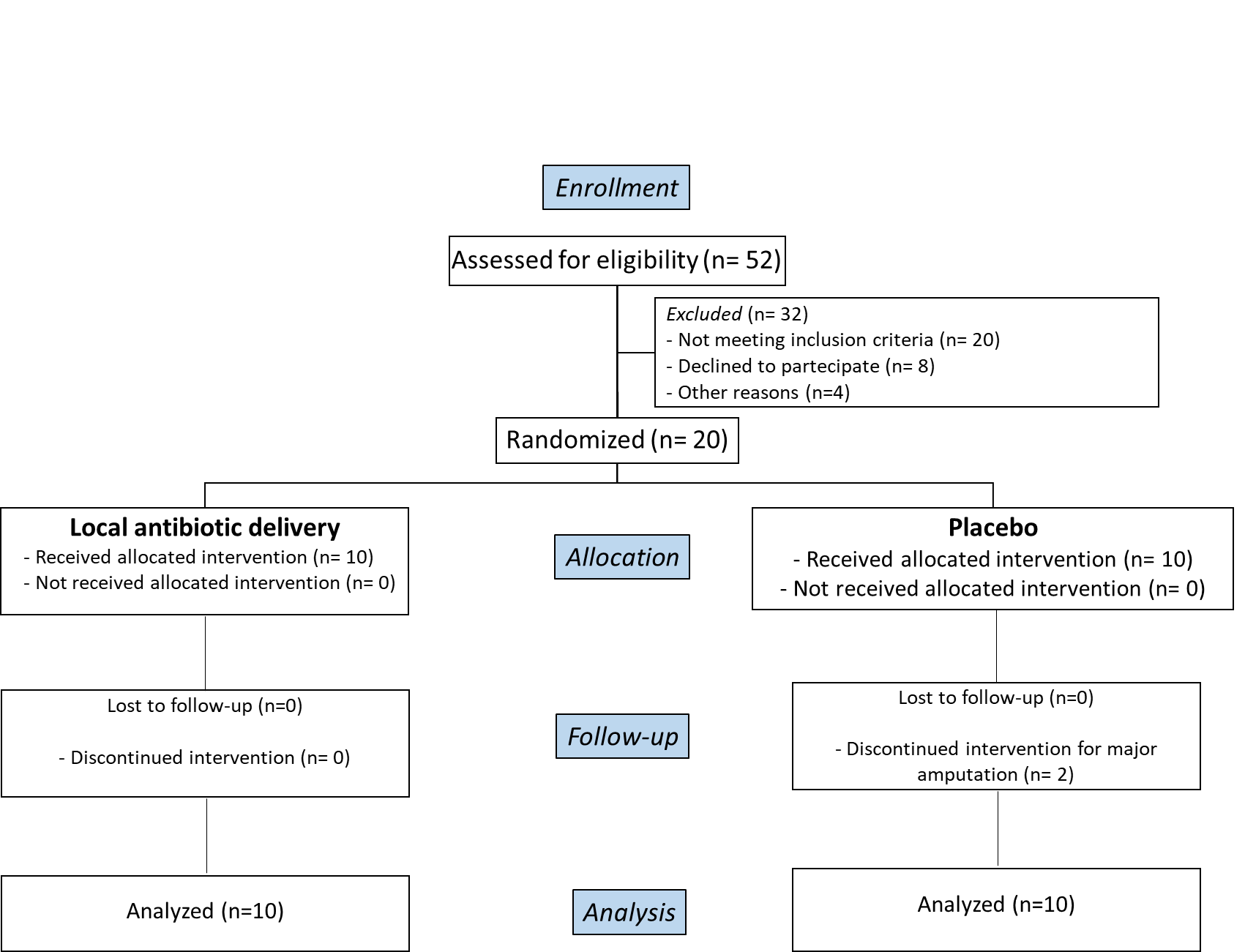

Supplement: Supplementary file 1 — DATA S1: Supporting Information. [file DOM-27-2552-s001.docx]
